# A New Survival Score for Patients ≥65 Years Assigned to Radiotherapy of Bone Metastases

**DOI:** 10.3390/cancers14194679

**Published:** 2022-09-26

**Authors:** Dirk Rades, Cansu Delikanli, Steven E. Schild, Charlotte Kristiansen, Søren Tvilsted, Stefan Janssen

**Affiliations:** 1 Department of Radiation Oncology, University of Lubeck, 23562 Lubeck, Germany; 2 Department of Radiation Oncology, Mayo Clinic, Scottsdale, AZ 85259, USA; 3 Department of Oncology, Vejle Hospital, University Hospital of Southern Denmark, 7100 Vejle, Denmark; 4 Research Department, Zealand University Hospital, 4600 Køge, Denmark; 5 Medical Practice for Radiotherapy and Radiation Oncology, 30161 Hannover, Germany

**Keywords:** bone metastases, elderly patients, radiation therapy, treatment personalization, survival score

## Abstract

**Simple Summary:**

Many cancer patients with bone metastases receive palliative radiotherapy. The patients’ remaining lifespan should be considered to achieve optimal treatment personalization. Since elderly patients (≥65 years) are different from younger ones, a specific survival score was developed for this age group. In a test cohort (*n* = 174), three prognostic groups were designed with 6-month survival rates of 0%, 51%, and 100%. The score was validated in another 174 patients. Moreover, the new score was compared to an existing tool developed in patients of any age. Compared to the previous tool, the new score was more accurate in predicting death ≤6 and ≤12 months and survival for ≥6 and ≥12 months. This demonstrates the importance of specific survival scores for the group of elderly patients.

**Abstract:**

Survival scores are important for personalized treatment of bone metastases. Elderly patients are considered a separate group. Therefore, a specific score was developed for these patients. Elderly patients (≥65 years) irradiated for bone metastases were randomly assigned to the test (*n* = 174) or validation (*n* = 174) cohorts. Thirteen factors were retrospectively analyzed for survival. Factors showing significance (*p* < 0.05) or a trend (*p* < 0.06) in the multivariate analysis were used for the score. Based on 6-month survival rates, prognostic groups were formed. The score was compared to an existing tool developed in patients of any age. In the multivariate analysis, performance score, tumor type, and visceral metastases showed significance and gender was a trend. Three groups were designed (17, 18–25 and 27–28 points) with 6-month survival rates of 0%, 51%, and 100%. In the validation cohort, these rates were 9%, 55%, and 86%. Comparisons of prognostic groups between both cohorts did not reveal significant differences. In the test cohort, positive predictive values regarding death ≤6 and survival ≥6 months were 100% with the new score vs. 80% and 88% with the existing tool. The new score was more accurate demonstrating the importance of specific scores for elderly patients.

## 1. Introduction

Up to 70% of patients with breast or prostate cancer and up to 40% of patients with kidney cancer develop bone metastases during the course of their disease [1,2,3]. Metastatic bone lesions represent the most frequent cause of cancer related pain [2]. Lesions located in the spine, the pelvis, or the base of the skull are often associated with intense pain [1,2].

Metastatic bone pain may increase over several weeks or even months. Patients typically describe their symptoms as burning pain with episodes of break-through pain and aggravation in the night [2]. These symptoms can often not be sufficiently controlled with administration of analgesic drugs. Therefore, many patients with painful uncomplicated lesions, i.e., lesions without (impending) fractures or spinal cord compression, receive radiotherapy alone. In case of (impending) pathological fractures or spinal cord compression, radiotherapy is often preceded by surgical stabilization [2,3]. In a large meta-analysis including 5617 patients, radiotherapy resulted in significant pain relief in approximately 60% of the patients [4]. Several dose-fractionation regimens are available for irradiation of bone metastases ranging from 8–10 Gy in one fraction to multi-fraction regimens lasting several weeks. Commonly used regimens include 1 × 8.0 Gy, 5–6 × 4.0 Gy, 10 × 3.0 Gy, 14–15 × 2.5 Gy and 18–20 × 2.0 Gy. Several meta-analyses of randomized trials showed that single-fraction radiotherapy as similarly effective as multi-fraction regimens in alleviating pain [4,5,6,7,8,9,10,11]. However, after longer-course regimens, a second course of radiotherapy due to recurrent pain was significantly less common. Moreover, in a randomized trial, recalcification of osteolytic bone was more pronounced after 10 × 3 Gy than after 1 × 8 Gy [12]. In a retrospective study, the biological effective dose of radiotherapy was the only independent predictor of increased bone density [13]. The evidence-based guideline of the American Society for Radiation Oncology (ASTRO) recommends single-fraction radiotherapy for painful uncomplicated bone metastases, particularly in patients with very limited survival prognoses. Since the stabilizing effect of radiotherapy often takes several months and recurrent bone pain occurs 6 months or later following radiotherapy, these aspects become more important with increasing lifetime. Thus, patients with bone metastases and favorable survival prognoses can benefit from multi-fraction radiotherapy with higher doses and likely from administration of bisphosphonates or denosumab to reduce the risk of skeletal-related events including pathological fractures, spinal cord compression and re-irradiation [2,3,14,15,16,17,18].

Therefore, for selection of the best possible dose-fractionation regimen, it is very important to be able to judge a patient’s survival prognosis prior to the start of treatment. To facilitate this judgement, several survival scores were developed for patients assigned to radiotherapy of bone metastases [19,20,21,22,23,24,25,26,27,28,29,30,31,32]. However, all but one of these scores were developed from data of patients treated until January 2008 or patients with motor deficits due to metastatic spinal cord compression, or limited to patients with spinal metastases or metastases of long bone. Moreover, only one of these survival scores was developed so far particularly for the group of elderly patients (≥65 years) [24]. However, that score was limited to patients with spinal cord compression leading to motor deficits. Thus, there is a need for a new survival score for elderly patients with bone metastases and without neurologic deficits. Specific prognostic scores for elderly patients appear important, since this age group usually faces more severe comorbidities and worse function of organs. Moreover, due to the demographic change, this age group is significantly growing [33]. Therefore, this study was performed in order to develop a survival score particularly for elderly cancer patients with bone metastases. Moreover, the new score was validated and compared to an existing tool developed in patients with bone metastases of different sites and no motor deficits but of any age treated between 2009 and 2017 [26].

## 2. Materials and Methods

A total of 348 elderly patients (≥65 years) irradiated for bone metastases without symptomatic spinal cord compression 2009–2021 were included in this retrospective study. Most common radiation regimen was 10 × 3 Gy over 2 weeks (equivalent dose in 2-Gy fractions = 32.5 Gy), which was used in 163 patients (47%). The entire cohort was randomly divided into a test cohort (*n* = 174) and a validation cohort (*n* = 174) using the excel random number generator. In the test cohort, the radiation dose given as equivalent dose in 2-Gy fractions (<32.5 Gy vs 32.5 Gy vs >32.5 Gy), the treatment period (2009–2017 vs. 2018–2022) and 11 potential prognostic factors were analyzed with respect to survival. These factors and their distributions in the test cohort and the validation cohort are shown in Table 1.

Univariate analyses were performed with the Kaplan–Meier method plus the log-rank test (BlueSky Statistics 10 GA, BlueSky Statistics LLC, Chicago, IL, USA). Significant factors (*p* < 0.05) in the test cohort were evaluated for independence with the Cox proportional hazards model. Factors achieving significance (*p* < 0.05) or showing a strong trend (*p* < 0.06) incorporated in the survival score. For each factor, 6-month survival rates were divided by 10. The resulting scoring points were added for each patient. Considering the 6-month survival rates of these patient scores, three prognostic groups were formed.

Subsequently, the three prognostic groups of the test cohort were compared to the corresponding groups of the validation cohort (Fisher’s exact test). Moreover, both cohorts were compared for accuracy to a previous scoring tool including also three prognostic groups, which was applied to the test and the validation cohort of the present study [26]. For these comparisons, the positive predictive values (PPVs) to correctly identify patients dying ≤6 months (worst prognostic groups) and patients surviving for ≥6 months (best prognostic groups).

The PPVs for correct prediction of death ≤6 months were calculated as follows:

PPV = [patients dying within 6 months/(patients dying within 6 months + patients not dying within 6 months)] × 100(1)

PPV = [patients surviving for 6 months/(patients surviving for 6 months + patients not surviving for 6 months)] × 100 (2)

## 3. Results

**Figure 1:**
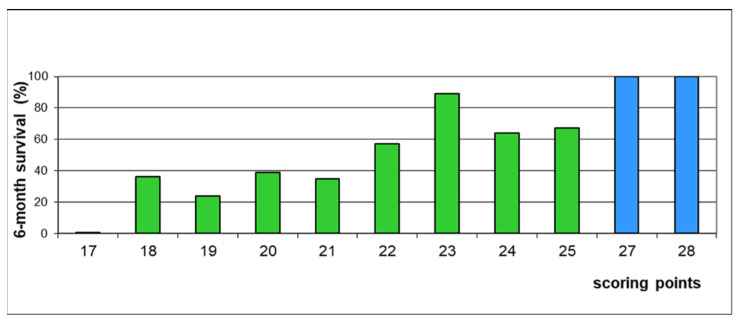
Scoring points for individual patients and corresponding 6-month survival rates.

**Figure 2:**
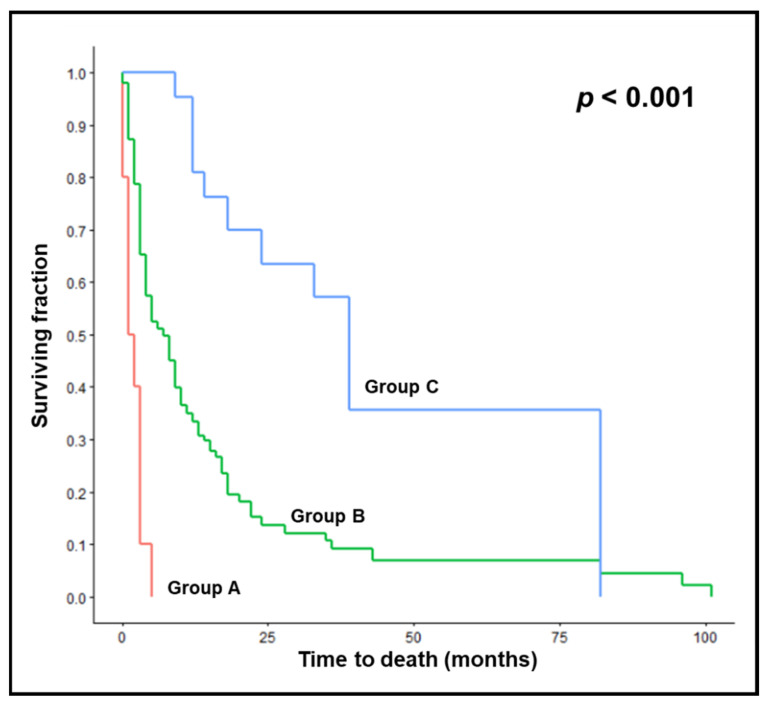
Test cohort: Kaplan–Meier curves for survival of prognostic groups A (17 points), B (18–25 points), and C (27–28 points). The *p*-value was calculated with the log-rank test.

On univariate analyses of the test cohort, female gender (*p* < 0.001), ECOG-PS 0–1 (*p* < 0.001), breast or prostate cancer (*p* < 0.001), and absence of visceral metastases (*p* = 0.009) were significantly associated with survival (Table 2). In the multivariate analysis, ECOG-PS (hazard ratio [HR] 2.30; 95% confidence interval [CI] 1.63–3.26; *p* < 0.001), primary tumor type (HR 1.10; 95% CI 1.02–1.19; *p* = 0.016), and visceral metastases (HR 1.58; 95% CI 1.10–2.27; *p* = 0.014) were significant, and gender (HR 1.46; 95% CI 0.99–2.15; *p* = 0.058) showed a strong trend. Therefore, all four factors were used for creating the survival score. The scoring points for these factors based on the 6-month survival rates are summarized in Table 3. Resulting individual patient scores ranged from 17 to 28 points (Figure 1). Considering the 6-month survival rates related to the patient scores, three prognostic groups were designed, i.e., 17 points (group A, *n* = 10), 18–25 points (group B, *n* = 141) and 27–28 points (group C, *n* = 23). No patient had 26 points. Median survival times of these groups were 1.5 months, 7 months and 39 months, respectively (*p* < 0.001). Survival rates were 0%, 51% and 100%, respectively, at 6 months, and 0%, 33% and 81%, respectively, at 12 months (Figure 2). Six-month survival rates were significantly different between groups A and B (Fisher’s exact test, *p* = 0.002) and groups B and C (*p* < 0.001).

**Figure 3:**
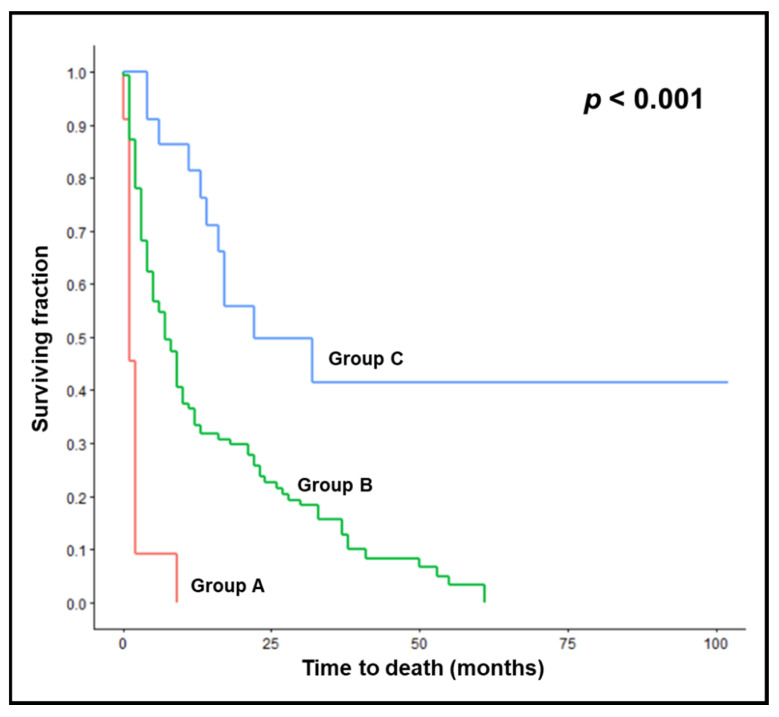
Validation cohort: Kaplan–Meier curves for survival of prognostic groups A (17 points), B (18–25 points), and C (27–28 points). The *p*-value was calculated with the log-rank test.

In the validation cohort, median survival times of prognostic groups A (*n* = 11), B (*n* = 141) and C (*n* = 22) were 1, 7 and 22 months, respectively (*p* < 0.001). Survival rates were 9%, 55% and 86%, respectively, at 6 months, and 0%, 33% and 81%, respectively, at 12 months (Figure 3). In the validation cohort, 6-month survival rates were also significantly different between groups A and B (*p* = 0.004) and groups B and C (*p* = 0.005). The comparisons of the prognostic groups between the test and the validation cohorts did not reveal significant differences between both groups A (*p* = 1.00, Fisher’s exact test), groups B (*p* = 0.63) and groups C (*p* = 0.11).

Subsequently, the new survival score was compared to an existing tool not specifically developed for elderly patients but for patients of any age [26]. In the test cohort, the PPVs of the new score to correctly identify patients dying ≤6 months and patients surviving for ≥6 months were 100% and 100%, respectively. When using the existing tool, the corresponding PPVs were 80% and 88%, respectively. When aiming to predict death ≤12 months and survival for ≥12 months, the PPVs were 100% and 81% with the new score compared to 89% and 70% with the existing tool. In the validation cohort, the PPVs of the new score to predict death ≤6 months and survival for ≥6 months were 91% and 86%, respectively, compared to 70% and 84%, respectively, with the existing tool. Regarding death ≤12 months and survival for ≥12 months, the PPVs were 100% and 81% with the new score, compared to 92% and 70% with the existing tool.

When considering these results, the new score was more accurate than the existing tool with respect to prediction of death ≤6 and ≤12 months and prediction of survival for ≥6 and ≥12 months. Therefore, the new score appeared preferable, which demonstrates the importance of separate prognostic tools for the group of patients aged ≥65 years.

## 4. Discussion

When selecting an individual treatment for patients irradiated for bone metastases, the survival prognosis plays an important role. Patients with poor expected survival should receive short treatment programs to avoid that they spend more than necessary of their remaining lifespan receiving cancer treatment. In contrast, for patients with more favorable prognoses late treatment-related side effects and long-term results become more important. This accounts particularly for metastatic disease and for elderly cancer patients, who may not be able to tolerate (aggressive) standard treatment regimens. Since different metastatic sites are associated with different prognoses, each site should be considered separately. Moreover, since many elderly patients have significant comorbidities in addition to their cancer disease and reduced function of organs, such as liver, kidney, and bone marrow, they would particularly benefit from personalized treatment regimens. In the present study, the first survival score was developed specifically for elderly patients (≥65 years) irradiated for bone metastases without neurological deficits due to metastatic spinal cord compression.

The new score includes three prognostic groups (A to C) with significantly different survival outcomes. In group A (poor prognosis) of the test cohort, the median survival time was only 1.5 months (validation cohort = 1 month), and all patients died within 5 months. Therefore, these patients should receive single-fraction radiotherapy (e.g., 1 × 8 Gy) in case of painful uncomplicated bone metastases or, otherwise, short-course radiotherapy (e.g., 5 or 6 × 4 Gy). This suggestion agrees with the recommendations of the ASTRO evidence-based guideline of radiotherapy for bone metastases [3]. These recommendations were based on several meta-analyses [4,5,6,7,8,9,10,11]. In these meta-analyses, single-fraction radiotherapy was not inferior to multi-fraction regimens with respect to pain relief, but re-treatment was significantly more frequent after single-fraction radiotherapy. However, recurrent bone pain requiring a second course of radiotherapy to the same area generally occurs only several months or even more than a year following irradiation [2,3]. Thus, patients of group A do not live long enough to be at a significant risk of recurrent bone pain.

Patients of group B (intermediate prognosis) in the test cohort had a median survival time of 7 months (validation cohort = 7 months). Approximately every second patient survived for ≥6 months, and approximately every third patient for ≥12 months. Therefore, re-irradiation and re-calcification of the osteolytic bone, which generally takes several months, has become more important [2,3]. Since in the several meta-analyses, re-irradiation was less common after multi-fraction radiotherapy, patients of group B may benefit from multi-fraction radiotherapy. Moreover, in a randomized trial of 107 patients from Germany, increase of bone density was significantly more pronounced after 10 × 3 Gy than after 1 × 8 Gy (173% vs. 120%, *p* < 0.001) [12]. Moreover, patients of this intermediate prognosis group may be considered for treatment with bisphosphonates (e.g., zoledronate) or a RANK-ligand inhibitor (denosumab). In a placebo-controlled trial of patients with metastatic hormone-refractory prostate cancer, zoledronate led to a significant reduction of skeletal-related events (33.2% vs. 44.2%, *p* = 0.021) [14]. Two-year rates of skeletal-related events were 28% and 49%, respectively (*p* = 0.028) [15]. In two randomized trials, the RANK-ligand inhibitor denosumab was significantly more effective in delaying the occurrence of skeletal-related events in patients with advanced breast cancer or castration-resistant prostate cancer [16,17]. In the placebo-controlled randomized trial investigating zoledronate, the median time to the first skeletal-related event in the placebo group was 321 days (i.e., 10.55 months) [14]. This shows that a survival time of at least several months is required to significantly benefit from administration of bisphosphonates or denosumab.

Patients of group C (favorable prognosis) in the test cohort had a median survival time of 39 months (validation cohort = 24 months). Moreover, 81% of these patients survived for ≥12 months and 63% for ≥24 months, respectively. Therefore, these patients should receive longer-course multi-fraction radiotherapy with higher doses to reduce the rate of re-irradiations and improve the increase in bone density [2,3,4,5,6,7,8,9,10,11,12]. In addition to the results of the studies and meta-analyses discussed above, the dose of radiotherapy (given as biologically effective dose) was the only factor significantly associated with increased bone density in the multivariate analysis of a retrospective study from 2021 [13]. This result supports the use of multi-fraction radiotherapy with higher doses (e.g., 15 × 2.5 Gy or 20 × 2 Gy) for patients with bone metastases and favorable survival prognoses. Moreover, patients of group C should strongly be considered for treatment with bisphosphonates or denosumab. In randomized trials, the median time to the first skeletal-related event ranged between 12 and >27 months, when these agents were given [14,15,16,17,18]. However, when using these agents, potential side effects including the 1–2% risk of osteonecrosis of the jaw should be considered.

When following these suggestions, the risk of a hidden selection bias due to the retrospective study design should be kept in mind. However, the score was validated within this study and proved to be superior to an existing score developed in patients of any age treated between 2009 and 2017 with respect to predicting death ≤6 and ≥12 months and survival for ≥6 and ≥12 months [26]. Therefore, the new score appears preferable for elderly patients ≥65 years assigned to radiotherapy of bone metastases without neurologic deficits caused by spinal cord compression. External validation in a prospective cohort of patients is warranted. A comparison to other existing tools was not performed, since all but one of these scores were created from patients treated until 1999, patients with symptomatic spinal cord compression, and/or patients with either spinal metastases or metastases of long bone only [19,20,21,22,23,24,25,26,27,28,29,30,31,32]. This held true also for the score of Westhoff et al., which although published in 2014 was based on data of patients treated between 1996 and 1998 [22], and the updated Katagiri score, which was also published in 2014, was developed in patients treated prior to February 2008 [32]. Since novel targeted therapies that can improve the survival of cancer patients have been increasingly used during the last 10 to 15 years, the new score was compared solely to a tool created from patients with bone metastases of any site mainly treated after 2010 [26,34,35,36,37].

## 5. Conclusions

A new survival score was created specifically for elderly patients (≥65 years) irradiated for bone metastases without motor deficits due to spinal cord compression. Given its limitations, this score achieved perfect (100%) accuracy in the test cohort with respect to correct identification of patients dying ≤6 months and patients surviving ≥6 months. In the validation cohort, PPVs were lower but still high (91% and 86%, respectively). Compared to a previous score developed in patients of any age, the new score was more accurate and, therefore, appeared preferable. Moreover, the new score can also be used to identify patients dying ≤12 months or surviving for ≥12 months. Ideally, the new score will be validated in a prospective cohort of patients.

## Figures and Tables

**Table 1:** Distribution of the potential prognostic factors in the test cohort (*n* = 174) and the validation cohort (*n* = 174).

Potential Prognostic Factor	Test Cohort *n* Patients (%)	Validation Cohort *n* Patients (%)	*p*-Value
Age			0.67
65–74 years	96 (55)	100 (57)	
≥75 years	78 (45)	74 (43)	
Gender			0.45
Female	79 (45)	72 (41)	
Male	95 (55)	102 (59)	
ECOG performance score			0.2
0–1	84 (48)	96 (55)	
≥2	90 (52)	78 (45)	
Primary tumor type			0.82
Breast cancer	45 (26)	38 (22)	
Prostate cancer	35 (20)	37 (21)	
Lung cancer	53 (30)	53 (30)	
Kidney cancer	8 (5)	11 (6)	
Colorectal cancer	8 (5)	4 (2)	
Cancer of unknown primary	6 (3)	8 (5)	
Other tumors	19 (11)	23 (13)	
Interval from tumor diagnosis to RT			0.33
≤8 months	78 (45)	87 (50)	
>8 months	96 (55)	87 (50)	
Visceral metastases			0.086
No	92 (53)	76 (44)	
Yes	82 (47)	98 (56)	
Other bone metastases			1
No	59 (34)	59 (34)	
Yes	115 (66)	115 (66)	
Upfront surgery			0.019
No	133 (76)	150 (86)	
Yes	41 (24)	24 (14)	
Pre-RT systemic therapy			0.36
No	51 (29)	59 (34)	
Yes	123 (71)	115 (66)	
Site(s) of irradiated lesions			0.84
Spinal only	65 (37)	60 (35)	
Non-spinal only	61 (35)	65 (37)	
Both	48 (28)	49 (28)	
Number of irradiated lesions			0.67
*n* = 1	80 (46)	76 (44)	
*n* ≥ 2	94 (54)	98 (56)	
Period of radiotherapy (years)			0.91
2009–2017	114 (66)	112 (64)	
2018–2022	60 (34)	62 (36)	
Radiotherapy dose (EQD2)			0.25
<32.5 Gy	20 (12)	26 (15)	
32.5 Gy (10 × 3 Gy)	89 (51)	74 (43)	
>32.5 Gy	65 (37)	74 (43)	

ECOG: Eastern Cooperative Oncology Group; RT: Radiotherapy; EQD2: Equivalent dose in 2-Gy fractions.

**Table 2:** Univariate analyses: Survival rates at 6 and 12 months of potential prognostic factors in the test cohort (*n* = 174).

Potential Prognostic Factor	Survival Rates at 6 Months (%)	Survival Rates at 12 Months (%)	*p*-Value
Age			0.37
65–74 years	50	31	
≥75 years	60	46	
Gender			<0.001
Female	65	49	
Male	46	28	
ECOG performance score			<0.001
0–1	73	57	
≥2	38	20	
Primary tumor type			<0.001
Breast cancer	78	59	
Prostate cancer	69	47	
Lung cancer	34	29	
Kidney cancer	50	13	
Colorectal cancer	50	13	
Cancer of unknown primary	33	33	
Other tumors	42	21	
Interval from tumor diagnosis to RT			0.4
≤8 months	46	36	
>8 months	61	39	
Visceral metastases			0.009
No	60	45	
Yes	49	30	
Other bone metastases			0.36
No	53	39	
Yes	56	37	
Upfront surgery			0.42
No	52	37	
Yes	63	41	
Pre-RT systemic therapy			0.38
No	47	32	
Yes	58	40	
Site(s) of irradiated lesions			0.74
Spinal only	58	38	
Non-spinal only	51	36	
Both	54	40	
Number of irradiated lesions			0.99
*n* = 1	54	40	
*n* ≥ 2	55	36	
Period of radiotherapy (years)			0.51
2009–2017	57	39	
2018–2022	50	36	
Radiotherapy dose (EQD2)			0.29
<32.5 Gy	55	25	
32.5 Gy (10 × 3 Gy)	56	44	
>32.5 Gy	52	32	

ECOG: Eastern Cooperative Oncology Group; RT: Radiotherapy; EQD2: Equivalent dose in 2-Gy fractions.

**Table 3:** Survival rates at 6 months and corresponding scoring points.

Characteristic	Survival Rate at 6 Months (%)	Scoring Points
Gender		
Female	65	7
Male	46	5
ECOG performance score		
0–1	73	7
2–3	38	4
Primary tumor type		
Breast cancer	78	8
Prostate cancer	69	7
Lung cancer	34	3
Kidney cancer	50	5
Colorectal cancer	50	5
Cancer of unknown primary	33	3
Other tumors	42	4
Visceral metastases		
No	60	6
Yes	49	5

ECOG: Eastern Cooperative Oncology Group.

## Data Availability

The data analyzed for this paper cannot be shared due to data protection regulations. According to the ethics committee, only evaluation of anonymized data is allowed for this study.
